# Supplementary material for: Causal Relationship between Aging and Anorexia Nervosa: A White-Matter-Microstructure-Mediated Mendelian Randomization Analysis
Source: Biomedicines. 2024 Aug 16;12(8):1874. doi: 10.3390/biomedicines12081874 (PMC11351342; doi:10.3390/biomedicines12081874)
Supplement: Supplementary file 1 [file biomedicines-12-01874-s001.zip › Supplementary Figure/Figure S4/Singe SNP plot of MR analysis for TL to ubm-b-2005.pdf]

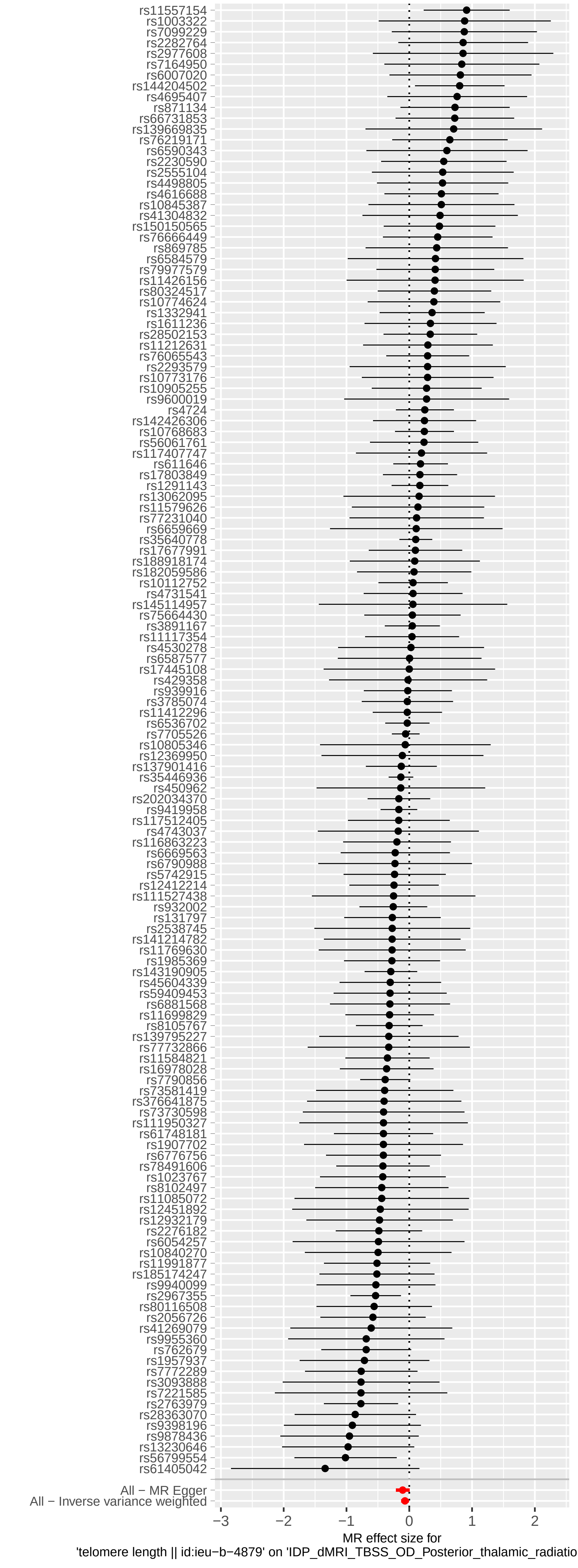
rs11557154
rs1003322
rs7099229
rs2282764
rs2977608
rs7164950
rs6007020
rs144204502
rs4695407
rs871134
rs66731853
rs139669835
rs76219171
rs6590343
rs2230590
rs2555104
rs4498805
rs4616688
rs10845387
rs41304832
rs150150565
rs76666449
rs869785
rs6584579
rs79977579
rs11426156
rs80324517
rs10774624
rs1332941
rs1611236
rs28502153
rs11212631
rs76065543
rs2293579
rs10773176
rs10905255
rs9600019
rs4724
rs142426306
rs10768683
rs56061761
rs117407747
rs611646
rs17803849
rs1291143
rs13062095
rs11579626
rs77231040
rs6659669
rs35640778
rs17677991
rs188918174
rs182059586
rs10112752
rs4731541
rs145114957
rs75664430
rs3891167
rs11117354
rs4530278
rs6587577
rs17445108
rs429358
rs939916
rs3785074
rs11412296
rs6536702
rs7705526
rs10805346
rs12369950
rs137901416
rs35446936
rs450962
rs202034370
rs9419958
rs117512405
rs4743037
rs116863223
rs6669563
rs6790988
rs5742915
rs12412214
rs111527438
rs932002
rs131797
rs2538745
rs141214782
rs11769630
rs1985369
rs143190905
rs45604339
rs59409453
rs6881568
rs11699829
rs8105767
rs139795227
rs77732866
rs11584821
rs16978028
rs7790856
rs73581419
rs376641875
rs73730598
rs111950327
rs61748181
rs1907702
rs6776756
rs78491606
rs1023767
rs8102497
rs11085072
rs12451892
rs12932179
rs2276182
rs6054257
rs10840270
rs11991877
rs185174247
rs9940099
rs2967355
rs80116508
rs2056726
rs41269079
rs9955360
rs762679
rs1957937
rs7772289
rs3093888
rs7221585
rs2763979
rs28363070
rs9398196
rs9878436
rs13230646
rs56799554
rs61405042
All − MR Egger
All − Inverse variance weighted
−3
−2
−1
0
1
2
MR effect size for
'telomere length || id:ieu−b−4879' on 'IDP_dMRI_TBSS_OD_Posterior_thalamic_radiatio
